# From Antibodies to Immunity: Assessing Correlates of Flavivirus Protection and Cross-Reactivity

**DOI:** 10.3390/vaccines13050449

**Published:** 2025-04-24

**Authors:** Hannah E. Flores, Eduar Fernando Pinzon Burgos, Sigrid Camacho Ortega, Alonso Heredia, Joel V. Chua

**Affiliations:** Division of Clinical Care and Research, Institute of Human Virology, University of Maryland School of Medicine, Baltimore, MD 21201, USA; hannahflores@ihv.umaryland.edu (H.E.F.); epinzonburgos@ihv.umaryland.edu (E.F.P.B.); scortega@ihv.umaryland.edu (S.C.O.); aheredia@ihv.umaryland.edu (A.H.)

**Keywords:** correlates of protection, Flaviviridae, Dengue virus, Japanese encephalitis virus, yellow fever virus, West Nile virus, Zika virus

## Abstract

Flaviviruses are arthropod-borne RNA viruses that can cause a wide range of human diseases, from mild symptoms to severe illness with multiorgan failure and death. Effective prevention of these diseases relies on identifying reliable vaccine targets, typically measured by correlates of protection (CoPs), which help indicate host immunity after vaccination. Current vaccines primarily focus on neutralizing antibodies (nAbs) against the viral envelope E protein, though emerging evidence suggests other potential targets may also be effective in disease prevention. Additionally, there is growing evidence of cross-protection between different flaviviruses when immunity to one virus is achieved, although this can be limited by antibody-dependent enhancement. This review examines the current understanding of flavivirus immunity, CoPs, and the potential for cross-protection in the context of existing vaccine strategies.

## 1. Introduction

Flaviviruses are RNA viruses that principally cause arthropod-borne disease, of either veterinary or human significance, or both [1]. Several pathogenic flaviviruses can cause significant morbidity and mortality in humans: dengue virus (DENV), Japanese encephalitis virus (JEV), West Nile virus (WNV), yellow fever virus (YFV), and Zika virus (ZIKV) [2,3]. These viruses are transmitted through vector-borne infections and produce a nonspecific syndrome consisting of the sudden onset of fever, chills, rash, and malaise. Yet, the severe state of the disease has unique clinical manifestations for each virus. Severe Yellow fever can progress to jaundice, bleeding, and shock [1,4]. DENV infection can progress to dengue hemorrhagic fever and dengue shock syndrome, which generally do not cause jaundice but may cause retroorbital pain, headache, and muscle pain [5]. JEV can cause acute encephalitis and neurologic signs, including seizures [6]. WNV can progress to encephalitis, meningitis, or neuroparalytic disease [7]. Finally, a Zika infection contracted during pregnancy has been associated with congenital microcephaly [8].

The global burden of human disease caused by flaviviruses is enormous. DENV alone is estimated to infect 400 million people annually, contributing to significant morbidity and mortality worldwide [9]. The flaviviruses addressed in this review are of major public health concern due to their epidemic and pandemic-prone nature [10,11]. While efforts exist to reduce the burden of disease in affected areas, there is still much work to be done to prevent current and future outbreaks, including the development of safe and effective vaccines.

Flaviviruses are small enveloped single-stranded positive-sense RNA viruses with a diameter ranging from 40–60 nm and a genome that is about 11 kilobases long [11,12]. The viruses share similar virion properties, structure, and genome organization but vary in their biological and antigenic properties [12]. Flavivirus virions contain multiple copies of the capsid (C) protein in complex with the single-stranded RNA molecule. The viral genetic material is released into the cytoplasm and translated to form the viral polyprotein. The polyprotein is then cleaved by viral proteases into the structural proteins E (envelope), C (capsid), and prM/M (premembrane/membrane) and nonstructural proteins NS1, NS2A/B, NS3, NS4A/B, and NS5 [13]. The nucleocapsid is enclosed within a host cell-derived lipid bilayer, which anchors 180 copies of the other two structural proteins, M and E, in a mature virion. The bilayer has an icosahedral layer organization, consisting of 90 E dimers [14].

**Figure 1:**
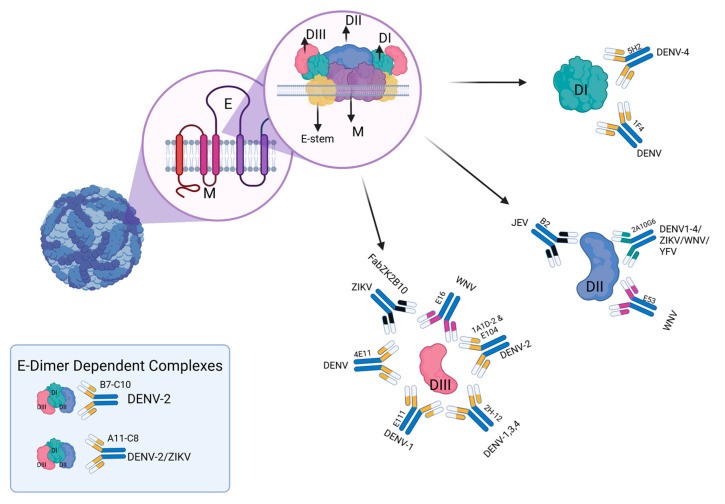
Complex structures of neutralizing antibodies bound target viral envelope (E) protein domains. The E protein consists of three domains DI, DII, and DIII, indicated in green, blue, and red, respectively. These domains are bound to the viral membrane by a stem of the protein E, shown in yellow, and two transmembrane domains of protein M, shown in purple. Along with the structure of the viral structure, we are presenting several complex NAbs-Flavivirus reported, some of these antibodies can recognize more than one dimer, or even some can recognize more than one flavivirus. DII: B2 [16], 2A10G6 [17], E53 [18]. DI: 5H2 [19], 1F4 [20]. DIII [21]: FabZK2B10 [22], E16 [23], 1A1D-2 [24], E104, 2H-12 [25], E111 [26], 4E11 [27] E-Dimer dependent: B7, C10 [28], A11 [28,29], C8 [28,30]. MAb: monoclonal antibody. The illustration was created by the author using Biorender.com, https://app.biorender.com/illustrations/67362c9f55c0d6e68341bced, accessed on 22 April 2025.

Flavivirus E protein attaches to cellular receptors and facilitates membrane fusion, followed by virion entry into target cells. This protein, which consists of three domains (Domain I or DI, Domain II or DII, and Domain III or DIII), is the major target of neutralizing antibodies (nAbs) that inhibit these functions and prevent infection (Figure 1). Neutralizing antibodies can mediate long-term protection from disease and are generally considered to be the best correlate of flavivirus immunity [2]. Highly neutralizing antibodies normally bind to the epitopes on the E protein, which are readily accessible and exposed on the virion surface. The effector function of humoral immunity against viruses is provided by these nAbs, which depend on the antibody concentration and avidity. For an antibody to be effective, it must reach the minimum neutralizing stoichiometry; approximately 30 antibodies are needed to occupy a viral particle. Factors that can affect the interaction between the neutralizing antibody and the viral epitope include the accessibility of the viral epitopes, the antibody concentration, and the binding affinity [15].

Efforts toward vaccine development are largely driven by the establishment of reliable vaccine targets. Correlates of protection (CoPs) are immune responses that function as targets for vaccine development. They are measurable biomarkers indicating that the immune system has responded to a threat. In some cases, these biomarkers may confer protection against future threats. CoPs include binding antibodies, neutralizing antibodies, and other components of the immune response [31]. Vaccines can vary widely in their efficacy and durability, and it is essential to understand the factors that drive vaccine-induced immunity and protection [32,33]. The breadth of mechanisms conferring immunity is incompletely understood, but it is well established that nAbs induced by viral vaccines are measurable and confer protection [32]. More robust activation of multiple arms of the immune response is generally considered to be more efficacious in vaccine development, although it has historically proven challenging to reliably measure cellular response. Advancements in measuring CD4+ and CD8+ T-cell memory seem a promising way forward, in addition to newer technology, such as bioengineering and virus-like particle vaccination [33].

The development of vaccines targeted against these viruses is not without adversary. One hurdle is antibody-dependent enhancement (ADE), where prior exposure to one flavivirus can lead to more severe disease upon subsequent infection with a different virus or serotypes [34]. This phenomenon, combined with the genetic diversity among flaviviruses, complicates vaccine design [34,35]. Ideally, vaccine strategies could capitalize on these immune responses to promote cross-protection rather than enhancement. Leveraging antibody cross-reactivity to boost both humoral and cellular immunity, while standardizing immune assays to define protection thresholds, is crucial for guiding future vaccine development.

Knowledge of vaccine targets against arboviruses is essential to mitigate the public health risk of large outbreaks. Currently, nAbs against E protein are the primary focus of vaccination efforts, although other targets may exist. This review aims to summarize the current understanding of flavivirus immunity, discuss the role of neutralizing antibodies in assessing vaccine efficacy, and evaluate how cross-reactivity can influence vaccine efforts.

## 2. Methods

A comprehensive literature review was conducted using PubMed to identify relevant studies that address correlates of protection in flavivirus infections. The search strategy involved the use of the following Boolean terms: “flavivirus” OR “Flaviviridae” OR “dengue” OR “Japanese encephalitis” OR “yellow fever” OR “west nile” OR “zika” AND “correlate of protection”. This search was performed on 4/15/2024, and no restrictions were applied regarding publication date or article type. The retrieved articles were screened for relevance, and studies that focused on immunological correlates, protective responses, or vaccine efficacy related to the aforementioned flaviviruses were included in the review.

## 3. Current Understanding of Correlates of Protection Against Flaviviruses

### 3.1. Dengue Virus (DENV)

Dengue is caused by four genetically and antigenically distinct DENV serotypes (DENV-1 to DENV-4), with homology of 65–70% [36]. DENV immune responses can either be protective or pathogenic. Following primary DENV infection, homotypic (same serotype) immune protection is long-lasting, whereas heterotypic (different serotype) protection is short-lived, generally lasting from several months to up to two years [37]. Waning heterotypic immunity is thought to play a significant role in a more intense DENV outbreak following the introduction of a second serotype in a population. In addition, different mutations in DENV proteins have generated new clades. For example, in Yucatan and Quintana Roo clades in the Mexican coastal region, genetic and phylogeographic analysis suggest the circulation of a lineage with mutations in the NS2A and NS5 proteins of DENV [38]. Although this new viral genetic evolution has had a limited impact on the tropism and cytopathogenicity of DENV, it may have modified the antigenicity of DENV strains, altering anti-DENV immune response in some patients. This alteration of immune responses could favor the development of severe forms of infection and dengue outbreaks, as seen in India, where nAbs against DENV serotypes 1, 3, and 4 decrease their neutralizing capacity against DENV with the same serotype but foreign strains [36,39].

Neutralizing antibodies are needed for protection and viral clearance; however, the magnitude of these antibodies may vary depending on the infecting serotype and the number of previous infections, whether natural or acquired via immunization [40]. The importance of neutralizing and sub-neutralizing antibodies during natural infections in the pathogenesis of severe presentation of DENV has been described since 1983 [41]. Two important immunologic phenomena are believed to occur: original antigenic sin and antibody-dependent enhancement (ADE).

Original antigenic sin occurs in heterotypic secondary infections where preexisting DENV immunity is elicited during a remote primary DENV infection of different serotypes. This phenomenon has been found to be biased against the heterotypic dengue virus, potentially resulting in a non-neutralizing event [41]. However, a higher mean titer of preinfection cross-reactive nAbs (1:260) was still observed to reduce the probability of experiencing symptomatic secondary infections down to 10% [42]. Additionally, high titer of nAbs were associated with a lower risk of developing a subsequent symptomatic disease [40]. However, nAb titers are not uniform, and other studies report reduced incidence of severe disease even with nAb titers > 1:40 [43]. High levels of nAbs in DENV infection depend not only on the host immune system, but also on the infecting serotype, with protection against secondary symptomatic infections in DENV-1 and DENV-2, but not DENV-3 [40].

In the second phenomenon, known as ADE, pre-existing cross-reactive (but non-neutralizing) antibodies paradoxically worsen disease through Fcγ receptor-mediated mechanisms. When these antibodies bind to new dengue serotypes, they form virus-IgG complexes that get internalized by phagocytes. This entry pathway: (1) enhances viral replication by bypassing normal immune clearance; (2) triggers a double-hit immunosuppression—simultaneously suppressing critical antiviral defenses (type-1 IFN, IL-12, nitric oxide) while promoting TH2-skewing cytokines (IL-6, IL-10); and (3) creates a perfect storm for immunopathology. The monocyte becomes a viral replication factory, the cytokine balance shifts toward ineffective responses, and the resulting vascular leakage manifests as severe dengue [44,45].

The E protein is organized into three discrete domains, envelope domain I (EDI), EDII, and EDIII [46]. EDI participates in conformational changes required for virus entry. EDII has a fusion loop (FL), which is required for fusion with the host cellular membrane and subsequent release of the DENV RNA into the cytosol. EDIII is believed to be responsible for interaction with the host-receptor molecule [47]. Most nAbs target serotype-specific epitopes (EDIII), generating a neutralizing response with low ADE-inducing power [48]. In contrast, highly cross-reactive antibodies, capable of recognizing prM and the EDII FL region of all four DENV serotypes, are poorly neutralizing but potent ADE promoters [49]. An analysis of memory B-cell responses in DENV-infected individuals showed that about 60% of the human antibody response is directed toward the prM protein, revealing that this protein and the FL epitope are particularly immunodominant and elicit potentially disease-enhancing cross-reactive antibodies [50].

In addition to the natural protection provided by nAbs, vaccines can help mitigate the occurrence of clinical manifestations and hospitalizations. Currently, the only U.S. FDA licensed dengue vaccine is Sanofi Pasteur’s live-attenuated tetravalent CYD-TDV vaccine (Dengvaxia^®^), consisting of a nonstructural backbone of yellow fever virus strain 17D, serotype-specific by replacing the prM and E regions of DENV-1-4 [51]. The overall vaccine efficacy ranges from 56.5% to 60.8% against all four DENV serotypes after the third dose of the vaccine and during a follow-up period of 13 months [52]. Serotype-specific protection against DENV-3 and DENV-4 was over 70% (DENV-4 77%-RR 0.23), while protection against DENV-1 and DENV-2 was less effective at 30–50% (DENV2 34%-RR 0.66) [53]. For all four DENV serotypes, neutralizing antibody geometric mean titers (GMTs) were highest for DENV-1 (176.59), DENV-2 (294.21), DENV-3 (258.78), and DENV-4 (189.35) in seropositive children after the third vaccine dose. In seronegative children 2–5 years of age, lower titers (GMT < 100) were associated with a more than seven-fold increase in the risk of hospitalization 1–2 years after vaccination [51,54,55]. A possible contributing cause is the lack of neutralizing antibodies and CD8 T-cell immune responses against nonstructural proteins, leading to reduced protection and durability observed for CYD-TDV [56].

TAK-003, also known as Qdenga vaccine, developed by Takeda, is also a live attenuated tetravalent dengue vaccine, including three chimeric viruses containing prM and E proteins of either DENV-1, 3, or 4, inserted into the DENV-2 backbone [57]. Clinical trials demonstrated that the vaccine had high efficacy against DENV-2 (95% GMT 1:1403.3), modest efficacy against DENV-1 (70% GMT 1:141.7) and less effective against DENV-3 (49% GMT 1:73.1); however, efficacy against DENV-4 (GMT 1:63.5) was inconclusive in the adult population (18–60 years). In the pediatric population (Table 1), titers were lower, yielding a cumulative efficacy of 61.2%, with an overall vaccine efficacy in preventing hospitalization due to dengue fever was 90%. The vaccine delivers DENV antigens to antigen-presenting cells and, through MHC class I, activates naive CD8+ T cells in the lymph nodes. It elicits CD8+ responses to NS1/NS3, which correlates with protection against DENV-2 [58]. When CD4+ and CD8+ T-cell responses were evaluated, they were observed to be multifunctional, inducing IFN-γ, TNF-α, and IL-2+ expression that persisted up to 3 years post-vaccination [59,60].

Another vaccine in late-phase clinical development is the Butantan dengue vaccine (Butantan-DV). This vaccine is a tetravalent, single-dose, live attenuated, full-length, tetravalent vaccine with one or more deletions in the 3′ untranslated region, the fourth component is a chimeric virus in which the prM and E proteins of DENV-2 replace those of DENV-4 in the DEN4Δ30 background [76]. In a Phase 3 trial, seroconversion for each virus was for DENV-1 (87%), DENV-2 (92%), DENV-3 (76%) and to DENV-4 (89%), with a significantly higher GMT in DENV-seropositive participants than in non-exposed participants in the Butantan-DV group for DENV1-3, but not for DENV-4 [63].

The induction of nAbs in DENV disease has been a challenge due to cross-reactivity between different serotypes, age, and serological status of the population. In pediatric and DENV-naive populations, nAb titers typically do not exceed 1:100, which is linked to lower vaccine efficacy. One possible explanation for the partial failure of quadrivalent dengue vaccines against certain serotypes (often DENV-3/4) is linked to Original Antigenic Sin, where pre-existing immunity, either from a previous infection or the vaccine itself, biases responses toward immunodominant serotypes (e.g., DENV-1/2). This bias occurs because memory B/T cells preferentially reactivate against familiar epitopes, suppressing robust responses to less dominant serotypes in the vaccine. As a result, neutralizing antibody and T cell responses become unbalanced, leaving gaps in protection against heterotypic serotypes. Furthermore, cross-reactive but non-neutralizing antibodies can facilitate ADE, exacerbating breakthrough infections. Therefore, Original Antigenic Sin weakens quadrivalent vaccine efficacy by creating hierarchical immunity rather than uniform protection across all four serotypes.

Additionally, there is no unified nAb measurement assay; the standardization of neutralization assays has required rigorous control of reagents (cell lines, viral strains, complement), methods (SOPs, controls), and interlaboratory harmonization using well-characterized reference panels. The performance of the plaque reduction neutralization test (PRNT), the world health organization (WHO) standard, with patient sera (acute/convalescent, primary/secondary, serotype-specific infections) under blinded and standardized conditions reveals significant variability in titers, influenced by the number of viral passages, cell line selection, and complement use [77]. MN has been used in high-throughput screening; however, it may overestimate cross-neutralization due to the cytopathic effect. The complex interactions between these factors affect reproducibility, which leads to inconsistent interpretation and analysis.

### 3.2. Yellow Fever Virus (YFV)

The yellow fever virus causes severe visceral involvement in approximately 20% of infected individuals with hepatic tropism. Mortality due to YFV occurred in 30% of those infected, associated with systemic shock, multi-organ failure, and immunopathology [78]. A YFV vaccine has been widely used in South America and Africa since 1937, when Max Theiler and colleagues generated the live attenuated YFV-17D vaccine by subculturing the Asibi virus 235–240 times for 3 years in mouse embryonic cells [69]. After injection into macaques, loss of virulence was observed, and antibodies were detected up to 12 weeks. In humans with previous exposure to YFV, post-vaccination titers after 4 weeks ranged from 1:8 to 1:125, but were 1:10 in those without prior exposure [69].

World War II veterans vaccinated with YFV-17D were assessed for persistence of vaccine-generated immunity 30–35 years after vaccination. Thirty-eight percent of vaccinees had high nAb titers (>1:64), while 68% had titers >1:8, and only 16.4% had negative titers (<1:2). These findings demonstrated the lifelong immunity conferred by the vaccine [70]. In a more recent study addressing the durability of YFV vaccinations, post-vaccination PRNT values remained positive but showed a decrease in nAb titer over time. Post-vaccination GMTs at 1-, 1.5-, 5-, and 10-years were 1046 (PRNT titer 1:160–2560), 452 (PRNT titer 1:160–640), 25% with PRNT titers < 100, and 35% with PRNT titers < 100, respectively [71,72]. Gutuzzo et al. demonstrated the longevity of the YFV vaccine through a systematic review in 2013, which prompted the WHO to conclude that a single dose of YFV-17D vaccine provided lifelong protection and that a booster dose was not necessary [79,80]. More recent literature attributes this vaccine efficacy to its ability to induce multiple arms of innate, cellular, and humoral immunity, with high levels of nAbs and memory T-cells detectable 10–60 years following vaccination [80,81,82]. This unique immunogenicity profile translates into superior vaccine efficacy and longevity [82].

To define post-vaccination protection, the cut-off point for PRNT seroconversion is established at antibody titers ≥ 1:10, or log neutralization index (LNI) ≥ 0.7, based on the antibody titer necessary to protect against lethal challenge in non-human primates [83,84]. This titer is associated with protective immunity, but it would be valuable to assess the efficacy of fractional YF vaccine dosing in cases of vaccine shortage, such as during an outbreak [85]. Additionally, applying different strategies is required in cases where YF vaccination is less clear, such as pregnancy, malnutrition, and people living with HIV, where vaccinating only in patients treated with antiretroviral therapy and with CD4+ T-cell counts above 350 cells/μL is the standard of care [86]. These special populations may still benefit from boosters, given limited evidence to suggest a reduction in immunity over time [81].

While the described vaccine strategy is widely touted as extraordinary due to its ability to induce long-term immunity, efforts toward YFV vaccination persist. Additional vaccine platforms being explored include mRNA vaccines and virus-like particles [87,88].

### 3.3. Japanese Encephalitis Virus (JEV)

JEV causes Japanese Encephalitis (JE), a severe neurological disease in humans that leads to intracranial swelling, manifested as high fever, headache, myalgias, and seizures. JEV primarily affects children and has a fatality rate between 20–30%, with morbidity up to 50% [89,90]. The WHO estimates 68,000 clinical cases of JE annually. The disease has no cure, making prevention a priority [91].

In the 1980s, virologists in China developed the live attenuated SA 14-14-2 vaccine. In 2005, the immune CoP was defined, expanding efforts toward JEV vaccine development [90]. The 4 main types of vaccines against JEV include inactivated mouse brain-derived vaccines, inactivated Vero cell-derived vaccines, live attenuated vaccines, and live recombinant (chimeric) vaccines [2,89,91]. All commercially approved vaccines have been developed against genotype III (GIII) strains of JEV [92]. The live attenuated SA 14-14-2 vaccine remains the primary childhood JE vaccine throughout Asia and the Western Pacific [93]. 

Like YFV, humoral immunity with the production of nAbs is considered the primary CoP for JEV [94]. These nAbs, primarily against the structural E protein and PrM, provide protective immunity [95,96]. This protective immunity is measured by PRNTs, the most specific serological test for differentiating flavivirus infections and the reference method for assessing the protective immune response following vaccination [97]. Serum nAb titers of greater than or equal to 1:10 are generally considered protective against JEV, and this titer is used as the cutoff for vaccine efficacy in clinical trials [98,99,100]. NS1, NS2A, and NS2B have also been investigated as vaccine targets with some success, although the protective epitope remains unclear [101]. Most recently, Kim et al. investigated a bivalent vaccination approach, showing that mice inoculated with GIII and GV antigens demonstrated superior protection against challenges from both GIII and GV JEV strains [92]. 

### 3.4. West Nile Virus (WNV)

WNV, a neurotropic flavivirus, is the leading cause of mosquito-borne and epidemic encephalitis in the United States [102]. It can cause several diseases, ranging from an acute febrile illness manifesting as headaches, myalgia, polyarthritis, rash, and lymphadenopathy, to more complicated central nervous system diseases like meningitis, encephalitis, or flaccid paralysis [102,103].

Studies in murine models, such as C57BL/6, have begun to elucidate the immune CoP against WNV infection. These CoPs require both innate and adaptive immune responses [104,105]. Currently, only four veterinary vaccines are approved for use on the market. Three of these are based on whole inactivated virus formulations, while one is a live chimeric virus vaccine [106,107]. All four vaccines require a two-dose primary immunization series followed by annual booster vaccinations to maintain protective immunity [108].

To date, no vaccines for West Nile virus (WNV) have been licensed for human use; however, six vaccine candidates have progressed to clinical evaluation. Among these, two candidates have advanced to Phase 2 clinical trials, including ChimeriVax-WN02, developed by Sanofi Pasteur in 2009 [109]. This candidate is based on a live-attenuated yellow fever 17D (YFV-17D) virus backbone and incorporates WNV structural genes. In clinical studies, ChimeriVax-WN02 elicited robust immune responses, with seroconversion rates ranging from 92% to 95% as measured by PRNT_50_ at 28 days post-vaccination. Importantly, no serious adverse events were reported during the trial [110].

The second candidate that progressed to Phase 2 evaluation is a formalin-inactivated WNV vaccine, which demonstrated a favorable safety profile and immunogenicity, as assessed by microneutralization. However, limited published data are available regarding this trial, and detailed information on the vaccine’s immunogenicity and safety remains unavailable [111].

In February of 2025, a new clinical trial was initiated by the Infectious Diseases Clinical Research Consortium (IDCRC) to evaluate the safety and immunogenicity of a second-generation inactivated WNV vaccine, HydroVax-001B (ClinicalTrials.gov identifier: NCT06745921). The study is designed to enroll healthy male and female adults between 18 and 49 years of age [112].

There is ongoing research regarding the innate immunological response for protection against WNV. Those studies have begun to identify the innate immune signaling network and antiviral effector genes that control WNV infection and protection [104,105].

In 1957, Porterfield and colleagues demonstrated that pretreatment of cells with Interferon (IFN) or the addition of IFN at the time of the infection inhibited WNV replication [113]. In agreement, a recent case report showed that two patients with WNV infection improved after 2 weeks of therapy with IFN-α2b [114]. At present, IFN dosing remains ill-defined but is a promising therapy for conferring protection in humans [115,116].

Monoclonal antibodies (MAbs), specifically against E protein, represent another promising area of research in our armamentarium against WNV [117]. Antibody protection against WNV might be through direct neutralization, complement activation, or increased viral uptake by phagocytic cells [118]. Regardless of the exact mechanism, MAbs like E16 have been recognized as a potent neutralizing antibody that neutralizes WNV strains with PRNT_50_ values of 4–18 ng [117].

The role of neutralizing IgM antibodies against WNV is also well established. In murine models, it was confirmed that mice with high anti-WNV Ig M titers (>1:45) on day 4 after infection had an improvement of at least 50% in survival rate versus mice who displayed lower antibody titers. Notably, all mice with low anti-WNV IgM titers (<1:45) on day 4 of infection succumbed [67]. 

On the other hand, IgG may confer some protection against WNV as well. In murine models, infected mice that received Intravenous immunoglobulin (IVIG) had no detectable virus in the blood or brain after two days of treatment, while mice who did not receive IVIG succumbed to infection at day 11 [68]. This protection is due to synergy between direct neutralization of receptor binding, Fc-receptor dependent viral clearance, complement-mediated lysis of virus or infected cells, and antibody-dependent toxicity [67]. 

CD8+ T cells have demonstrated a role in protective immunity against WNV. Although the mechanism of this discordant lymphocyte population is under investigation, preliminary data suggest higher mortality in mice exhibiting a CD8+ T cell deficiency [119]. Nevertheless, it has been shown that CD4+ T cells are also an important CoP, due to their production of IFNγ during acute WNV infection, which may aid in viral clearance. In fact, CD4+ T cell-deficient mice also show increased mortality during WNV infection [120]. 

Interest in the role of CD4+ T cells in the immune response to WNV infection has grown in recent years, primarily due to their critical function in providing help to B cells for the generation of neutralizing antibodies, which represent the principal CoP in flavivirus infections [121].

Moreover, it has been demonstrated that CD4+ T Cells can confer protection in lethally infected RAG-/- mice, independent of both B cells and CD8+ T Cells. These WNV -specific CD4+ T cells not only produce significant amounts of IFNγ but also secrete substantial levels of IL-2. Furthermore, they exhibit notable cytotoxic potential, both in vivo and ex vivo cytotoxicity, highlighting their critical role in antiviral immunity beyond traditional antibody and CD8+ T cell-mediated response [122].

The pivotal role in orchestrating adaptive immune responses against WNV is demonstrated by the fact that dominant CD4+ T helper cell responses are directed primarily toward two α-helical regions in the viral capsid and to exposed domains of the E protein [121]. This immunodominance pattern closely mirrors observations made in other flaviviruses such as DENV and ZIKV, highlighting conserved mechanisms of immune recognition within this viral family [123,124]. Such targeted responses are critical not only for supporting antibody production but also for shaping long-term immunity through the development of high-affinity memory B cells and durable humoral protection [121].

Memory B cells and long-lived plasma cells generated during WNV infection persist over time, continuing to secrete high-affinity antibodies specific to immunodominant epitopes in the viral envelope protein (E) of both wild-type and mutant WNV strains. Notably, some of these cells produce antibodies targeting specific epitopes to which they have never been previously exposed. This observation suggests that these memory B cells, and plasma cells may provide a mechanism for neutralizing potential future challenges posed by WNV variants, offering insight into the adaptive immune system’s capacity for cross-protection against viral diversity [125].

The role of B cells in long-term protection against WNV is further highlighted by a study using C57BL/6J-(BAFFR)^-/-^ mice, which exhibit a profound reduction in mature B cells but retain normal numbers of newly formed, immature B cells [126]. These mice were able to control WNV infection following passive transfer of immune serum from previously infected wild-type mice. Notably, a similar protective effect was observed when a vaccine was administered, provided the formulation could program immature B cells to produce virus-specific antibodies. This induced long-term protection was sustained for up to 60 days post-challenge, emphasizing the critical contribution of antibody-mediated immunity (even in the absence of fully mature B cell populations) to durable protection against WNV [127].

### 3.5. Zika Virus (ZIKV)

The WHO declared Zika viral infection a Public Health Emergency of International Concern in February of 2016 [128]. In most cases, the infection has been associated with febrile illness and episodes of rash, arthralgia, and conjunctivitis [129]. The epidemic registered in 2016 was also linked to congenital Zika syndrome (CZS), causing Guillain-Barre Syndrome, microcephaly, and congenital malformation, particularly in pregnant people infected during the first trimester [130]. While transmission occurs through the bite of an infected mosquito, it can also be transmitted through blood transfusions, sex, and vertically through delivery.

ZIKV induces both innate immune and adaptive immune responses in the host. IFN response is crucial to control the infection. Several studies have shown that the virus is sensitive to the antiviral effect of both type I and type II IFNs [131]. In mouse models, ZIKV proteins, such as NS5, are capable of inhibiting IFN signaling through the degradation of antiviral transcriptional activator STAT2 [132]. Moreover, it has been demonstrated that ZIKV replicates better in mice lacking IFN α/β receptors, highlighting the relevance of the IFN-mediated response during the infection [133]. 

Neutralizing antibodies against viral epitopes on ZIKV envelope protein dimer or in EDIII seem to be the most important response for protection. Antibodies isolated from previously ZIKV-infected subjects have been shown to protect mice from both mosquito-borne infection and maternal-fetal transmission [134,135]. 

CD4+ and CD8+ T cells are critical in the host response against ZIKV-infected mice, responsible for producing both effector cytokines and cytolytic molecules during infection [136]. This response is effective independent of IFNγ-mediated effects [137]. Additionally, murine models that lack CD4+ T cells have been shown to develop fewer ZIKV specific nAbs [138].

Despite several promising vaccine candidates tested in preclinical models, there remains no clear consensus on the minimum nAb titer required for protective immunity. Reported protective thresholds vary widely across studies. Dowd and colleagues estimated that the titers required for protection in macaques and mice was 1:100 (using microneutralization (MN) assays). In contrast, Pardi and colleagues, using mRNA vaccines, showed the nAb titers required were closer to 1:1000, likely reflecting differences in vaccine platform, immune kinetics, and the stringency of the challenge model [75,139].

In human clinical trials, even greater variability exists. For instance, a phase I trial using an inactivated virus vaccine reported that titers > 1:10 were associated with protection based on MN assays [73]. These discrepancies likely stem from differences in assay format (e.g., PRNT vs. MN), viral strains, host species, host immune background, immunization route, and challenge conditions. This underscores the challenge of establishing a universal CoP for ZIKV. Further clinical studies with standardized assay methodologies and harmonized endpoints will be necessary to determine the precise nAb titers required for protective immunity in humans across vaccine platforms.

Multiple ZIKV vaccine candidates, including DNA-based, mRNA, inactivated, and viral vector platforms, have demonstrated immunogenicity and protective efficacy in these preclinical models and have advanced to early-phase clinical trials. Among these, the DNA vaccine GLS-5700, developed by Inovio Pharmaceuticals and GeneOne Life Science, was the first ZIKV vaccine to enter a Phase 1 trial, demonstrating safety and induction of neutralizing antibodies in humans [140]. The mRNA vaccines mRNA-1325 and mRNA-1893, developed by Moderna, also progressed into Phase 1 clinical evaluation. mRNA-1893 showed strong immunogenicity and advanced to a Phase 2 study [141,142]. In addition, ZPIV, an inactivated whole-virus vaccine developed by the Walter Reed Army Institute of Research, was tested in multiple Phase 1 clinical trials with various adjuvant combinations [73].

Despite these developments, none of these vaccine candidates has received regulatory licensure for human use. The lack of widespread ZIKV outbreaks has limited the ability to conduct large-scale efficacy trials, while uncertainties around standardized neutralizing antibody thresholds and CoP continue to present regulatory challenges [143]. Continued clinical evaluation and harmonization of immunological endpoints remain essential to advancing a licensed ZIKV vaccine.

## 4. Cross-Reactive Immunity Between Flaviviruses

Due to the high degree of sequence identity shared among flaviviruses, cross-reactive responses can be protective or promote immunopathology. Structurally, flaviviruses share a high degree of homology. High-resolution electron density maps have shown that the mature ZIKV structure is similar to mature DENV and WNV structures, with only a 10-amino-acid difference surrounding the Asn154 glycosylation site found in the E protein [144]. Most of the nAbs recognize the viral E protein, which is the major glycoprotein in the surface of the virions and whose DII interacts with the membranes of the cell during fusion [145].

In fact, now we know that the DENV nAbs recognize highly conserved residues at the FL of DII in the E protein. Most antibodies are cross-reactive but non-neutralizing against heterologous serotypes, with only a minor proportion having neutralization activity [146]. Specificity of WNV antibodies against the conserved residues in DII of the viral E protein has also been characterized both in human and murine models [147,148].

Relative to the cellular response, CD4+ and CD8+ T-cell responses to ZIKV are stronger in DENV-positive individuals. Grifoni and colleagues provided evidence of ZIKV/DENV cross-reaction in human peripheral blood cells and suggested that DENV-specific memory T-cells recognize peptide sequences located in the ZIKV proteome. The average homology between DENV and ZIKV cross-reactive epitopes reaches at least 77%, explaining the cross-reactive response, which predominantly targets structural proteins [149].

Although the activation of the cells secondary to a DENV infection has been identified, it is not yet clear if this response is protective during a ZIKV infection. Despite extensive cross-reactivity in IgG binding, evidence for ZIKV neutralization has been limited, and, when present, it disappears after 6 months [81]. One study showed at least 88% E protein-specific cross-reactivity, yet only one sample showed potent neutralization. This lack of consistency in conferred protection against ZIKV from primary DENV infection may be explained by the timing of sample collection in these studies, which are typically collected during the acute phase of infection [150].

Moreover, the choice of animal models is an important factor to consider. In a macaque model, the efficacy of a purified inactivated Zika virus vaccine was evaluated in animals with pre-existing immune responses to YFV, JEV, and WNV. This study found that prior immunity to YFV or DENV did not affect the generation of ZIKV-neutralizing antibodies following challenge. However, a limitation of the study was the absence of a contemporaneous unvaccinated control group challenged in parallel with the vaccinated cohorts, which may partially account for the observed results [151]. However, it is likely that other factors impact the production of proper cross-reactive neutralization antibodies among flaviviruses.

Cross-reactive antibodies are also capable of forming virus-antibody complexes, which, through ADE, can worsen symptoms during secondary infection, particularly in DENV and JEV [152,153,154]. In the presence of DENV mAbs, infection by ZIKV is likewise enhanced [150]. Mouse models have also been used to demonstrate the ADE effect, and it has been shown that certain antibodies targeting domains I/II of the E protein against ZIKV can cross-react with DENV and exacerbate disease in infected mice [155].

This effect has also been demonstrated using a panel of human MAbs derived from DENV-infected individuals, showing that most antibodies targeting the DENV envelope protein also cross-react with ZIKV. Furthermore, the study showed that antibodies specific to linear epitopes, including the immunodominant fusion loop epitope, were capable of binding ZIKV but failed to neutralize the virus, instead promoting ADE [156].

In vivo, this effect has also been reported using convalescent plasma from DENV-infected individuals in ZIKV-susceptible mice. Bardina and colleagues demonstrated significant enhancement of ZIKV infection, with treated mice exhibiting increased morbidity, including fever, viremia, and mortality. Notably, this study also reported ADE following administration of serum from WNV-infected individuals, highlighting the potential for cross-reactive flavivirus antibodies to exacerbate ZIKV pathogenesis [157].

While this phenomenon can lead to severe consequences in naturally infected individuals, it may also be utilized to enhance vaccine-induced responses. For example, individuals with cross-reactive antibody titers from a virus inactivated JEV vaccination had enhanced YFV immunogenicity after YFV vaccination. This response induced greater pro-inflammatory responses, potentially conferring enhanced protection [158].

This effect has also been observed in the serum of individuals vaccinated against JEV, who exhibited the ability to neutralize WNV. The close antigenic relationship among flavivirus is further underscored by the finding that this cross-neutralizing response was enhanced in certain recipients with previous vaccination against YFV as well [11,159].

Taken together, these findings highlight the dual nature of cross-reactive flavivirus immunity, offering both opportunities for cross-protection and challenges related to potential immunopathology, particularly through mechanisms such as ADE.

The immunological cross-reactivity among flaviviruses has sparked interest in the development of pan-flavivirus vaccines capable of providing broad protection. Both memory B cells and T cells have demonstrated cross-reactive potential, particularly in the context of vaccine candidates currently under investigation for protection against DENV and ZIKV. In vivo studies have confirmed cross-protection against both viruses, and safety evaluations have reported no evidence of hepatotoxicity in immunized mice [160].

Most of the broadly neutralizing antibodies target conserved epitopes on the envelope protein. As shown in Figure 1, some of these antibodies are capable of neutralizing multiple flavivirus species and could serve as the basis for epitope-focused vaccine strategies [161].

Finally, conserved E-dimer epitopes are gaining attention for their ability to elicit robust and specific immune responses, making them promising targets for pan-flavivirus vaccine development. In murine models, E-dimer constructs incorporating domain I (EDI) and domain II (EDII) have been shown to induce virus-specific neutralizing IgG responses at levels approximately threefold higher than their monomeric counterparts. These findings suggest that the stabilized quaternary structure of the E-dimer enhances the immunogenic presentation of conserved epitopes, thereby promoting the induction of cross-reactive antibody responses across flavivirus species [162].

Notably, memory T cell responses to ZIKV peptides have been observed following prior infection with DENV or immunization with tetravalent live-attenuated dengue vaccines. This cross-reactivity is attributed to the high degree of sequence conservation between DENV and ZIKV peptides recognized by DENV-elicited memory T cells. Furthermore, the quality of the T cell response appears to be modulated by the frequency of DENV-specific CD8^+^; T cells in individuals with prior DENV exposure. These cells have been shown to selectively upregulate granzyme B and PD-1, a phenotype not observed in DENV-naïve donors [149].

Nevertheless, it is challenging because most are poorly neutralizing and could contribute to the ADE effect, raising safety concerns [163]. Nonetheless, advances have been made to use B cells to produce potently neutralizing antibodies against previously unknown but promising epitopes [164].

## 5. Conclusions

The Flaviviruses covered in this review (DENV, YFV, JEV, YFV, WNV, and ZIKV) have the potential to cause epidemic infection in humans. While symptomatic care is the mainstay of treatment, prevention is a key pillar of care. Safe and effective vaccines exist for YFV and JEV, but additional work is needed to further enhance preventative measures. Current vaccination efforts primarily utilize nAbs against the flavivirus structural proteins, but the role of non-structural proteins as potential targets should not be overlooked. Ideally, leveraging antibody cross-reactivity to further enhance vaccine immunogenicity by invoking both humoral and cell-mediated immunity is an important area of investigation for future vaccine endeavors. Furthermore, defining host immunity is imperative to define vaccination schedules and determine the duration of protection, but this is limited in the setting of non-standardized assays and titer interpretation, which vary by lab. Predicting a clinically useful threshold of protection against each flavivirus is essential to prove the efficacy of vaccination and establish a basis to drive vaccine policy preceding and during epidemics.

## Figures and Tables

**Table 1:** Flaviviruses and Corresponding Correlates of Protection.

Disease Caused by Flaviviruses	Potential Correlate of Protection	Evidence	Reference
**Dengue**	Higher levels of nAb titer Anti-DENV (1:260) reduce by 10% the risk of symptomatic DENV infection in pediatric cohort (2–14 y). (Technique: PRNT 50 *)	Higher median pre-infection nAb titer is associated with a reduced likelihood of symptomatic DENV infection in endemic areas. Short time intervals between primary and secondary infection are associated with protection from clinical manifestations.	[42]
	CYD-TDV/Dengvaxia: Pediatric seropositive patients (2–16 y): GMT were DENV1 1:274, DENV2 1:445, DENV3 1:384, and DENV4 1:196.7, with an efficacy of 83.7%. (Technique: PRNT 50)	A better correlation of neutralizing titers was observed for DENV3 and 4 compared to the other serotypes. In the seronegative population, GMTs were low: DENV1 1:35.3, DENV2 1:105, DENV3 1:93.6, and DENV4 1:89.5, with no correlation with protection (43.2%).	[52,61]
	TAK-003/Qdenga Pediatric seronegative patients (4–16 y): GMT were DENV1 1:87.8, DENV2 1:929.4, DENV3 1:71.7, and DENV4 1:64 with an efficacy of 66.2%. (Technique: MN 50 **)	Compared to seronegative children, seropositive children had an efficacy of 76.1%, which increased titers up to 3-fold, with an efficacy of 95.1% for DENV2.	[59,60,62]
	TV-003/TV-005/Butantan-DV In the seronegative population (2–59 y), the GMTs were DENV1 1:280.7, DENV2 1:167-5, DENV3 1:114-6, and DENV4 1:151-1, and the seropositive population was higher (DENV1 1:841.7, DENV2 1:732.3, DENV3 1:299.8, and DENV4 1:238.0), yielding an efficacy of 79.6%. (Technique: PRNT 50)	A correlation of GMT with protection was observed in all age ranges; the lowest efficacy was 64.6% in children aged 2–6 years, increasing to over 70% in all other age groups.	[63,64]
**Japanese B encephalitis**	There is no efficacy data for JE-VC. However, the titer of ≥10 is an established immunologic correlate of protection. (Technique: PRNT 50)	Phase III, multicenter, multinational, observer-blinded trial with 867 adults included, which proved seroconversion rate in 98% of the volunteers vaccinated with JE-VAX^®^	[65,66]
**West Nile viral encephalitis**	Anti-WNV IgM titers of ≥1:45 (Technique: PRNT 50)	Correlated with improved survival rate in WNV-infected C57BL/6J mice The absence of secreted IgM resulted in 100% lethality in response to WNV infection in mice. The survival rate for mice with titers of ≥ 1:45 of anti-WNV Ig M was 70% at day 4 of the infection.	[67]
	Anti-WNV IgG titers of 1:1600 to 1:3200 (Technique: ELISA)	Conferred 100% protection on BALB/c mice when challenged with WNV. BALB/c mice inoculated intraperitoneally with 100 pfu or 1000 pfu of WNV were protected from the infection using passively transferred mouse anti-WNV hyperimmune serum with titers >1:1600.	[68]
**Yellow fever**	Patients’ previous exposure to YFV; 4 weeks post-vaccination titers ranged from 1:8 to 1:125 (Technique: PRNT 50)	YFV-17D, 1937	[69]
	Subjects without exposure; 4 weeks post-vaccination titers were 1:10 (Technique: PRNT 50)		
	NT50 titers of >1:64 (37.9%) >1:8 in 68% and 16.4% were negative (<1:2) (Technique: PRNT 50)	Persistence of YFV-17D vaccine at 30–35 years	[70]
	Years post-vaccination; 1 year GMT 1046 (PRNT50 titer 1:160–2560), 1.5 GMT 452 (PRNT titer 1:160–640) at 5 and 10 years the PRNT titers < 100 (Technique: PRNT 50)	Persistence of YFV-17D vaccine	[71,72]
**Zika viral disease**	Anti-ZIKV neutralizing antibody titers of >1:10 (Technique: MN 50) Anti-ZIKV neutralizing antibodies titers between >1:10 and >1:100 was evaluated (Technique: PRNT 50)	Phase 1, placebo-controlled, double-blind trial that tested a purified formalin-inactivated Zika virus vaccine. At day 57 post vaccine, volunteers had an 88% seroconversion rate (nAbs titer of 1:10) and GMT titer against ZIKA virus of 100. The U.S. Army developed an inactivated vaccine candidate using a 2015 Puerto Rican ZIKV Strain. human volunteers were vaccinated, and their polyclonal IgG was purified and infused into mice at varying concentrations challenged with ZIKV. These mice experienced complete or partial protection from RNAemia following challenges.	[73,74]
	Anti-ZIKV neutralizing antibodies titers of ≥1300 (in BALB/C mice) and ≥400 (in Rhesus macaques) (Technique: PRNT 50)	Preclinical trial that studied the effect of a single immunization with nucleoside-modified ZIKV prM-E mRNA-LNP vaccine in eliciting durable protection against detectable viremia in mice and rhesus macaques. Titers ≥400 protected 80% of Rhesus Macaques against infection with WNV, whereas titers ≥1300 protected 100% of detectable viremia in BALB/c mice challenged with ZIKV.	[75]

Abbreviations: DENV (dengue virus); PRNT 50 (plaque reduction neutralization test); CYD-TDV (Chimeric Yellow Fever Virus-DENV Tetravalent Dengue Vaccine); GMT (geometric mean titer); MN 50 (mean neutralizing antibody titer); WNV (West Nile Virus); YFV (Yellow Fever Virus); NT50 (neutralization test 50%); ZIKV (Zika Virus). * PRNT 50: The reciprocal of the serum dilution that reduces viral plaques by 50%. ** MN 50: The reciprocal of the serum dilution that inhibits 50% of virus-induced cytopathic effect in a microneutralization assay.
